# Langerhans Cell Histiocytosis (LCH): Guidelines for Diagnosis, Clinical Work-Up, and Treatment for Patients Till the Age of 18 Years

**DOI:** 10.1002/pbc.24367

**Published:** 2012-10-25

**Authors:** Riccardo Haupt, Milen Minkov, Itziar Astigarraga, Eva Schäfer, Vasanta Nanduri, Rima Jubran, R Maarten Egeler, Gritta Janka, Dragan Micic, Carlos Rodriguez-Galindo, Stefaan Van Gool, Johannes Visser, Sheila Weitzman, Jean Donadieu, for the Euro Histio Network

**Affiliations:** 1 Department of Hematology and Oncology, Epidemiology and Biostatistics Section, Istituto G. GasliniGenova, Italy; 2 Children's Cancer Research Institute, St. Anna Children's HospitalVienna, Austria; 3 Hospital Universitario Cruces BarakaldoBarakaldo, Spain; 4 Reference Centre for Histiocytosis at Hopital Trousseau, Assistance Publique – Hopitaux de ParisFrance; 5 Watford General HospitalWatford, UK; 6 Children's Hospital of Los AngelesLos Angeles, California; 7 Hospital for Sick ChildrenToronto, Ontario, Canada; 8 University Medical Center Hamburg-EppendorfHamburg, Germany; 9 Mother and Child Health Institute of Serbia “Dr Vukan Cupic,” BelgradeSerbia; 10 Dana Farber Cancer Institute, Children's Hospital BostonBoston, Massachusetts; 11 University Hospital GasthuisbergLeuven, Belgium; 12 University Hospitals of Leicester, Leicester Children's HospitalLeicester, UK

**Keywords:** clinical work-up, diagnosis, follow-up, guidelines, Langerhans cell histiocytosis, therapy

## Abstract

These guidelines for the management of patients up to 18 years with Langerhans cell histiocytosis (LCH) have been set up by a group of experts involved in the Euro Histio Net project who participated in national or international studies and in peer reviewed publications. Existing guidelines were reviewed and changed where new evidence was available in the literature up to 2012. Data and publications have been ranked according to evidence based medicine and when there was a lack of published data, consensus between experts was sought. Guidelines for diagnosis, initial clinical work-up, and treatment and long-term follow-up of LCH patients are presented.

## INTRODUCTION

Langerhans cell histiocytosis (LCH) is a heterogeneous disease, characterized by accumulation of dendritic cells with features similar to epidermal Langerhans cells in various organs. Any organ or system of the human body can be affected, but those more frequently involved are the skeleton (80% of cases), the skin (33%), and the pituitary (25%). Other organs involved are the liver, spleen, the hematopoietic system and the lungs (15% each), lymph nodes (5–10%), and the central nervous system excluding the pituitary (2–4%). The clinical course may vary from a self-limiting disease to a rapidly progressive one that might lead to death. Between 30% and 40% of patients may develop permanent adverse sequelae. Treatment options vary depending on the extent of the disease and the severity at onset. Response to front-line treatment is an important information to adapt the therapeutic strategy. As LCH is a rare disease, only a limited number of large surveys or of randomized clinical trials are available in the literature and many aspects of the management of patients remain obscure or controversial. The presented guidelines are based on published evidence and the clinical expertise of the authors. They are intended to provide guidance with respect to diagnosis and clinical work-up of LCH occurring in patients <18 years old. The recommendations can neither replace the physician's own professional judgement nor consider all special clinical circumstances which may apply to individual cases.

## METHODS

This document is derived from the project Euro Histio Net 2008, a reference network (http://www.eurohistio.net) for LCH and associated syndromes in the European Union which received funding within the framework of the Public Health Program. The guidelines were designed and established by European and North American physicians considered to be experts in the field of pediatric histiocytic disorders. They are active members of the international medical society of histiocytoses “Histiocyte Society” (HS), of the European national societies of Hematology/Oncology, and of their respective national groups for the study and treatment of these diseases. The guidelines have been developed for use as recommended practice in the evaluation and treatment of children and teenagers up to 18 years with LCH. Scientific articles published in peer-reviewed journals up to January 2012 were systematically reviewed. In addition to the medical literature, the following guidelines are a synthesis of different international and national guidelines and recommendation documents.

Evidence was ranked in four levels [1, 2]: (A) meta-analyses, high quality systematic reviews, or randomized controlled trials with a low risk of bias; (B) systematic reviews of case–control or cohort studies; (C) non-analytic studies; for example, case reports, case series, small retrospective studies; (D) expert opinion. Level of agreement between experts and data was ranked in three classes: (2) general agreement between all experts or between available studies; (1) discussed recommendation, but no formal objections between experts or mild difference between studies, without contradiction for the main endpoint; (0) divergence of opinion or contradictory results for the main endpoint.

### Langerhans Cell Histiocytosis Diagnosis

Since LCH may affect any organ or system of the body, the condition should be considered whenever suggestive clinical manifestations occur in the skin, bone, lung, liver, or CNS. Table I shows a list of differential diagnoses to be considered depending on presenting complaints, signs, or symptoms. The diagnosis is clinicopathologic and should only be made in the appropriate clinical setting to prevent a misdiagnosis in the presence of normal reactive Langerhans cells, particularly in regional lymph nodes. In addition to clinical and radiological features, LCH diagnosis should always be based on histological and immunophenotypic examination of lesional tissue (agreement: 2), that should be taken from the most easily accessible, yet representative lesion.

**TABLE I:** Differential Diagnosis for Manifestations of Langerhans Cell Histiocytosis

Involvement	Manifestation	Possible other condition
Skin	Vesicles and bullae (most common in early infancy)	Erythema toxicum
		Herpes simplex
		Varicella
	Dermatitis (most frequently scalp, diaperarea, or axilla, may occur up to late infancy)	Seborrheic dermatitis (eczema; usually no petechiae and marked scaling)
	Nodules	Mastocytosis
		Juvenile xanthogranuloma
		Neuroblastoma
		Infant leukemia
	Pruritus	Scabies (other family members may be affected)
	Petechiae	
Bone	Vertebra plana	Ewing sarcoma
		Septic osteomyelitis
		Chronic relapsing multifocal osteomyelitis (CRMO)
		Leukemia
		Lymphoma
		Aneurysmal bone cyst
		Juvenile xanthogranuloma
		Myeloma (only described in adults)
		Osteoporosis
	Temporal bone	Chronic otitis media
		Mastoiditis
		Cholesteatoma
		Soft tissue sarcoma
	Orbit	Acute infection (preseptal cellulitis)
		Dermoid cyst
		Rhabdomyosarcoma
		Neuroblastoma
		Erdheim–Chester disease
		Pseudoinflammatory tumor
	Other lytic lesions of the long bones	Septic osteomyelitis
		Chronic recurrent multifocal osteomyelitis (CRMO)
		Aneurysmal bone cyst
		Bone angiomatosis (Gorham disease)
		Fibrous dysplasia
		Atypical mycobacterial infection
		Osteogenic sarcoma
		Ewing's sarcoma
Lung	In particular systemic symptoms and cavitated pulmonary nodules	Pneumocystis jirovecii cavitated infection
		Mycobacterial or other pulmonary infections
		Sarcoidosis
		Bronchiolar–alveolar carcinoma (only described in adults)
		Lymphangio–Leiomyosarcoma (only described in young adult women)
		Septic emboli
Liver	Jaundice with direct hyperbilirubinemia	Chronic destructive cholangitis
	Hypoalbuminemia	Metabolic disease
		Hepatitis
		Neoplasia obstructing biliary tract
		Inherited deficient conjugation of bilirubin
		Toxic (Reye syndrome)
		Chronic inflammatory bowel disease
		Neonatal hemochromatosis
Endocrine	Diabetes insipidus	Central nervous sysytem germ cell tumor
		Hypophisitis

There is a well defined histologically characteristic appearance of the LCH lesions on hematoxylin and eosin stained sections, but positive CD1a and/or CD207 (Langerin) staining of the lesional cells is required for a definitive diagnosis [3–6] (agreement: 2). Electron microscopy is no longer needed (agreement: 2), since it has been shown that the expression of Langerin correlates with the ultrastructural presence of Birbeck granules. Diagnostic confirmation may be a challenge in some circumstances (e.g., liver specimens), where Birbeck granules are not present and CD1a and/or Langerin may be negative because LCH cells have regressed after having caused sclerosing cholangitis and Cirrhosis [7].

In rare cases the risk of biopsy may outweigh the need for a definitive diagnosis, and therefore the risk/benefit ratio should be carefully assessed. This is the case in patients with isolated involvement of a vertebral body without an adjacent soft tissue component, as in case of vertebra plana, or with isolated involvement of the odontoid peg. If the decision to avoid or postpone a biopsy is made, every effort should be made to rule out other conditions that might lead to a similar radiological finding (Table I).

Patients without a histologically confirmed diagnosis need to be carefully monitored by appropriate imaging for at least the next 6 months in order to reassess the need for biopsy and its justification, in order to exclude a malignancy.

### Pretreatment Clinical Evaluation

Once the diagnosis of LCH has been ascertained it is important to collect further baseline information in order to decide on a therapeutic approach. A complete history should include special reference to the nature and duration of symptoms. Specific symptoms to be sought are: pain, swelling, skin rashes, otorrhea, fever, loss of appetite, diarrhea, poor weight gain, growth failure, polydipsia, polyuria, respiratory symptoms, irritability, behavioral, and neurological changes. A detailed examination should be performed at the onset and at each follow-up visit. Currently there is no specific biological marker of disease activity, however, there is a general agreement (agreement: 2) that biochemical and imaging evaluation at diagnosis and at disease reactivation should include the mandatory investigations listed in Table II. Certain scenarios might require additional testing; the recommended laboratory investigations, imaging, or specialized clinical assessments upon specific indication are shown in Table III; the detailed protocol for head MRI is provided in (Supplemental Appendix I).

**TABLE II:** Laboratory and Radiographic Evaluation of Children With LCH

Evaluation
Full blood count
Hemoglobin
White blood cell and differential count
Platelet count
Blood chemistry
Total protein
Albumin
Bilirubin
ALT (SGPT)
AST (SGOT)
γGT
Creatinine
Electrolytes
Erythrocyte sedimentation rate (ESR)
Abdominal ultrasound (in particular for young children)
Size and structure of liver and spleen
Abdominal lymph-nodes
Coagulation studies
INR/PT
APTT/PTT
Fibrinogen/factor I
Chest Radiograph (CXR)
Skeletal radiograph survey^a^,^b^

ALT (SGPT), alanine transaminase (serum glutamic pyruvic transaminase); APTT/PTT, activated partial thromboplastin time/partial thromboplastin time; AST (SGOT), aspartate transaminase (serum glutamic oxaloacetic transaminase); γGT, gamma-glutamyltransferase; INR/PT, international normalized ratio/prothrombin time; MRI, magnetic resonance imaging; PET, positron emission tomography; Tc, technetium.

a Note that other imaging techniques as bone Tc scan, PET scan, or MRI are not an alternative to the standard skeletal survey. The real value of these images in LCH is still under study. In particular information from bone scan should not be considered for evaluation of disease extent and decision-making. PET scan has proven to be the most sensitive functional test used in the identification of LCH lesions and in evaluating patient response to therapy. However, it is currently expensive, exposes the patient to a significant radiation dose and is not widely available [56].

b It is not recommended to change the method of bone evaluation (skeletal radiograph), as it may lead to discrepancy between assessments. It is important also to consider the ALARA principle (as low as reasonably achievable) for ionizing radiation and, if possible, during follow up, limit the evaluation to the anatomic region initially involved.

**TABLE III:** Specific Clinical Scenarios and Recommended Additional Testing in Children With LCH

Clinical scenario and recommended additional testing
History of polyuria or polydipsia
Early morning urine specific gravity and osmolality
Blood electrolytes
Water deprivation test if possible
MRI of the head^a^
Bicytopenia, pancytopenia, or persistent unexplained single cytopenia
Other causes of anemia or thrombocytopenia has to be ruled out according to standard medical practice. If no other causes are found, the cytopenia is considered LCH-related
Bone marrow aspirate and trephine biopsy to exclude causes other than LCH b as exposant
Evaluation for features of macrophage activation and hemophagocytic syndrome (triglycerides and ferritin in addition to the coagulation studies in Table IIa^c^
Liver dysfunction
If frank liver dysfunction (liver enzymes >5-fold upper limit of normal/bilirubin >5-fold upper limit of normal): consult a hepatologist and consider liver MRI which is preferable to retrograde cholangiography
Liver biopsy is only recommended if there is clinically significant liver involvement and the result will alter treatment (i.e., to differentiate between active LCH and sclerosing cholangitis)
Lung involvement (further testing is only needed in case of abnormal chest X-ray or symptoms/signs suggestive of lung involvement, or pulmonary findings not characteristic of LCH or suspicion of an atypical infection)
Lung high resolution computed tomography (HR-CT) or preferably low dose multi-detector HR-CT if available. Note that cysts and nodules are the only images typical of LCH; all other lesions are not diagnostic. In children already diagnosed with MS-LCH (see section “Clinical Classification”) low dose CT is sufficient in order to assess extent of pulmonary involvement, and reduce the radiation exposure
Lung function tests (if age appropriate)
Bronchoalveolar lavage (BAL): >5% CD1a + cells in BAL fluid may be diagnostic in a non-smoker^d^
Lung biopsy (if BAL is not diagnostic)
Suspected craniofacial bone lesions including maxilla and mandible
MRI of head^a^ including the brain, hypothalamus–pituitary axis, and all craniofacial bones. If MRI not available, CT of the involved bone and the skull base is recommended
Aural discharge or suspected hearing impairment/mastoid involvement
Formal hearing assessment
MRI of head^a^ or HR-CT of temporal bone
Vertebral lesions (even if only suspected)
MRI of spine to assess for soft tissue massess and to exclude spinal cord compression
Visual or neurological abnormalities
MRI of head^a^
Neurological assessment
Neuropsychometric assessment
Suspected other endocrine abnormality (i.e., short stature, growth failure, hypothalamic syndromes, precocious, or delayed puberty)
Endocrine assessment (including dynamic tests of the anterior pituitary and thyroid)
MRI of head^a^
Unexplained chronic diarrhea, failure to thrive, or evidence of malabsorption
Endoscopy
Biopsy

(HR-)CT, (high resolution) computed tomography; MRI, magnetic resonance imaging.

a See Appendix 1 for details.

^b^The clinical significance of CD1a positivity in the bone marrow remains to be proven. An isolated finding of histiocytic infiltration on the bone marrow with no cytopenia is not a criterion for diagnosis or reactivation [57, 58].

c Hemophagocytic syndrome with macrophage activation is a common finding in patients with hematological dysfunction [59, 60].

d See discussion in Refs. [12, 13].

### Defining Organ Involvement, Risk Organs, and CNS (Central Nervous System) Risk Lesions

The findings of the pretreatment clinical evaluation allow definition of organ involvement based on the clinical, biological, and radiological criteria shown in Table IV. Disease involvement of certain organs is considered as a marker of higher risk of (a) dying from disease (risk organs) or (b) developing neuro-degenerative complications more commonly named as CNS risk lesions.

**TABLE IV:** Definition of Organ Involvement in Langerhans Cell Histiocytosis

Criteria	CNS risk lesions	Risk organ
Bone involvement		
General bone involvement: all radiologically documented lesions, which are not mentioned below		
Craniofacial bone involvement: lesions in the orbital, temporal, mastoid, sphenoidal, zygomatic, or ethmoidal bones; the maxilla or paranasal sinuses; or cranial fossa; with intracranial soft tissue extension	Yes	
Vertebral involvement without soft tissue extension, for example, vertebra plana		
Vertebral involvement with intraspinal soft tissue extension or lesions in the odontoid peg		
An abnormality on Tc bone scan or an MRI hypersignal, not correlated with symptoms, or with an X-ray image is not considered bony disease!		
Central nervous system (CNS) involvement	Yes	
Tumoral: all intracerebral expansive lesions predominantly affecting the brain or meninges		
Neurodegeneration on MRI: MRI imaging compatible with neurodegenerative disease^a^, that is, abnormal signal intensity localized in the dentate nuclei or cerebellum or cerebral atrophy NOT explained by corticosteroids		
Clinical neurodegeneration: presence of suggestive symptoms (either cerebellar syndrome or learning difficulty) with compatible MRI imaging		
Ear involvement		
Ear involvement with external otitis, otitis media, or otorrhea	Yes	
Eye involvement		
Orbital involvement with proptosis or exophthalmos	Yes	
Hematopoietic involvement		Yes
Mild (both of the following categories should be present)		
Hemoglobin between 10 and 7 g/dl (not due to other causes, e.g., iron deficiency)		
Thrombocytopenia with platelets between 100,000 and 20,000/mm^3^		
Severe (both of the following categories should be present)		
Hemoglobin <7 g/dl (not due to other causes, e.g., iron deficiency)		
Platelets <20,000/mm^3^		
Liver involvement (the patient can show a combination of these symptoms)		Yes
Enlargement >3 cm below the costal margin at the mid clavicular line, confirmed by ultrasound or dysfunction documented by: hyperbilirubinemia >3 times normal hypoalbuminemia (<30 g/dl), γ GT increased >2 times normal, ALT (SGPT)–AST (SGOT) >3 times normal, ascites, edema, or intra hepatic nodular mass		
Lung involvement		(Yes)^b^
Typical imaging (nodules or cysts) on CT scan		
Any atypical mass needs to be explored by BAL or biopsy in order to have histopathological/cytological diagnosis		
Mucosa involvement		
Oral involvement with lesions in the oral mucosa, gums		
Genital or anal involvement		
Pituitary involvement		
Any pituitary hormone deficiency or tumor appearance in the hypothalamic-pituitary axis		
Skin involvement		
Any rash documented by histological examination or any lesion (erythematous and crusted macules, papules, or nodules, with or without ulceration, or petechiae, or seborrhea-like picture) compatible with the diagnosis, if LCH is confirmed by biopsy of another organ		
Spleen involvement		Yes
>3 cm below the costal margin at the mid clavicular line, confirmed by ultrasound		

ALT (SGPT), alanine transaminase (serum glutamic pyruvic transaminase); AST (SGOT), aspartate transaminase (serum glutamic oxaloacetic transaminase); BAL, bronchoalveolar lavage; CT, computed tomography; MRI, magnetic resonance imaging.

a The term radiological neurodegeneration has been coined to describe a certain pattern of MRI findings, but this terminology may be misleading as it does not necessarily correlate with histopathology.

b See section “Risk organs.”

Risk organs include the hematologic system, the spleen and the liver (evidence: B, agreement: 2) [8–10]. The lung had been considered for many decades as a risk organ, but its individual prognostic impact has recently been questioned [11]. In fact, in the absence of involvement of other risk organs, lung disease is only in exceptional cases the ultimate cause of death [12, 13], and this usually occurs through “mechanical complications” such as an uncontrolled pneumothorax [14], or as a late event due to chronic emphysematous changes. In the upcoming clinical trial for LCH in children (LCH-IV), the lung will be no longer considered a risk organ.

Involvement of some skull bones might predispose to diabetes insipidus (DI) and CNS manifestations [15–17]. The term CNS risk lesions, representing a more recent concept [16], suggests that these patients are more likely to develop neuro-degenerative CNS disease, which may be an irreversible complication of LCH and may have a debilitating course [18]. Therefore, skull bone lesions, with the exception of the vault, are considered as CNS risk lesions, assuming that risk factors for DI can also be considered as risk factors of neuro-degenerative changes (evidence C; agreement: 1).

### Clinical Classification

The current classification differentiates between single system disease (SS-LCH) and multisystem disease (MS-LCH), a distinction based on the extent of involvement at diagnosis. In SS-LCH, only one organ or system is involved such as bone (either as a single bone or more than one bone), skin, lymph node (not the draining lymph node of another LCH lesion), lungs, hypothalamic-pituitary/central nervous system, or others such as thyroid or thymus. In MS-LCH, two or more organs, or systems are involved either with or without involvement of risk organs.

### General Considerations for Treatment

#### Treatment of single-system LCH

Patients with SS-LCH may be initially referred to a range of medical specialists depending on the localization and presentation of the lesions, thus it is difficult to organize and execute coordinated international trials. This section combines the limited published evidence with the authors' experience (evidence: C, agreement: 1).

#### Single system unifocal bone involvement (isolated bone lesions)

Unifocal bone lesions are the predominant clinical form of LCH. Spontaneous regression may occur, and the clinical course is probably not greatly influenced by any form of treatment. The decision on the most appropriate approach should be based on clinical symptoms, the size and location of the disease, and on any evidence of healing on imaging. Often, simple curettage during the diagnostic biopsy will result in healing, and further intervention may not be necessary [19]. Indications for additional treatment include involvement of weight-bearing bones, imminent spinal cord compression, unacceptable deformity, intense pain, and functional disability.

Complete excision of bone lesions (curettage) may be indicated if the lesion is small (<2 cm) and is combined with the diagnostic confirmation. However, radical excision of large lesions (>5 cm) is not indicated since it increases the size of the bony defect, could prolong the time to healing, and might result in permanent skeletal defects. For lesions 2–5 cms in diameter, a biopsy and partial curettage is an option. Depending on the size and location of the lesion, an intralesional injection of methylprednisolone may be administered [20] to promote healing (evidence: C). Immobilization of the limb may need to be considered and discussed with the orthopedic surgeon in rare cases. “Vertebra plana” *per se* is not an indication for an orthopedic corset, and expert physiotherapy assessment should be considered; however, temporary immobilization may be required for symptomatic relief in the early phases of vertebral involvement. Patients with temporal bone lesions and recurrent otorrhea, may have a secondary cholesteatoma which may need specific treatment [21].

In certain functionally critical anatomical sites, such as the odontoid peg or other vertebral lesions with intraspinal soft tissue extension there may be an immediate risk to the patient because of the potential for disease progression and the hazards involved in attempting a biopsy; however, these are exceptional situations, and a biopsy should always be considered. Isolated disease involving functionally critical anatomical sites may justify systemic therapy.

Because of the potential for development of sequelae, systemic therapy is indicated in patients with lesions involving the skull base, temporal bone, orbits, and vertebral column, where there is also involvement of the adjacent soft tissues.

#### Single system multifocal bone involvement

LCH which presents with only multiple bone lesions at diagnosis (SS-LCH multifocal bone) usually remains confined to the skeleton, and only rarely extends to other organs like the skin and pituitary gland. However, the incidence of reported reactivations in cases of multifocal bone disease is higher than for unifocal bone disease [22–24]. Regardless of the treatment approaches that vary from observation only to systemic chemotherapy, survival rates approaching 100% are reported for this disease form in almost all the published series. Therefore, the benefit of therapies should be evaluated in terms of localization and length of disease activity, and hence, risk of permanent consequences and quality of life. Unfortunately, due to discrepancy in anatomic bone lesions and outcome assessment, published data are difficult to compare and therefore no definitive conclusions can be reached. The most commonly used therapy for multifocal skeletal LCH consists of steroids and vinblastine (VBL), a relatively non-toxic, and well tolerated combination.

#### Single system skin involvement (isolated cutaneous LCH)

LCH confined to the skin is rare and accounts for about 5% of the LCH population. It can occur at any age, but is most common in newborns and infants. In most of these cases LCH tends to regress spontaneously, but progression to MS-LCH is common. Therefore, close follow-up and reassessment of the need for treatment is warranted in all young patients with this disease form.

Cutaneous lesions can appear either as isolated nodules or as a skin rash; in patients with isolated nodules surgical excision may be indicated, but radical surgery is never warranted. In children with a skin rash, topical steroids are often suggested in standard textbooks, but their efficacy has never been proven. Moreover, most patients with isolated cutaneous LCH are often diagnosed after unsuccessful treatment with topical steroids for other presumed diagnoses such as eczema [25]. Topical caryolysine (20% nitrogen mustard ointment) has been shown to be effective on skin LCH [26]. Even with potential mutagenesis effect, no secondary tumor deleterious effect has been reported in relation to this drug for this indication. Unfortunately, it is not easily available and necessitates application by trained personnel.

In cases of ineffective local therapy or involvement of an extensive area, systemic therapy with steroids (±VBL), or oral low dose methotrexate can be used, but the level of evidence is low (D) [27–29]. In the most severe cases, treatments, including thalidomide associated with neurological toxicity, pain and fatigue [29], azathioprine, or PUVA-therapy which have been shown to be effective in some adult patients, might also be considered in children (evidence: D, agreement: 1).

### Single System LCH of the Lymph Nodes

This is an extremely rare presentation of LCH [30]. Excision biopsy may be the only treatment required for a solitary lymph node.

#### Single system LCH of the lung (primary pulmonary LCH)

This rare disease form occurs predominantly in adolescent and adult smokers. The impact of systemic therapy is not well documented in children, and adult pulmonologists do not consider it as the standard approach [12, 13]. Smoking withdrawal is necessary, and usually results in significant clinical improvement and often complete resolution. However, isolated lung involvement can be very challenging due to the risk of acute severe complications such as pneumothorax, or cardiopulmonary arrest. Pneumothoraces should be treated by standard techniques such as drainage and possibly pleurodesis. Pleurectomy should be avoided as lung transplantation may ultimately be considered in patients with severe progressive disease. In case of persisting and progressive lung disease, systemic therapy with low dose steroids is most commonly used, but 2-chlorodeoxyadenosine (2-CdA), and the combination of VBL and steroids have also been used (evidence: D, agreement: 1).

#### Isolated diabetes insipidus and pituitary involvement

DI occurs due to involvement of the posterior pituitary (neurohypophysis) and may become manifest either before, concurrently, or after LCH diagnosis. Isolated DI is not considered an indication for systemic therapy *per se*, except when active disease is unequivocally documented by the presence of thickening of the pituitary stalk or a mass lesion of the hypothalamic-pituitary axis. A lesion of the hypothalamus–pituitary axis is usually considered as active if it had local neurological consequences like alteration of the visual field or if its volume is increasing on sequential MRI. In the experience of experts, DI is with few exceptions uniformly irreversible, although DDAVP needs may vary.

There are some earlier anecdotal reports suggesting that treatment with 2-CdA [31], etoposide [32], or radiation [33, 34] soon after DI onset may reverse the condition (evidence: D).

#### Brain lesions

In addition to pituitary stalk lesions, any brain, or meningeal lesion (except local reaction to a skull vault lesion) is considered an indication for systemic therapy. The standard therapy with vinblastine and steroid can be effective in this situation [35] or 2-CdA monotherapy [36].

#### Treatment of multisystem LCH

As mentioned before, the major clinical challenges of MS-LCH are mortality in young children with involvement of risk organs, and bouts of reactivation resulting in morbidity and permanent consequences which can occur in all age groups. Patients with risk organ involvement are at risk of death, and a poor response to therapy defines a sub-group with a particularly dismal prognosis. Patients without involvement of risk organs, although not at risk for mortality, need systemic therapy in order to control the disease activity, reduce reactivations, and reduce permanent consequences. Several international protocols for MS-LCH treatment have been designed within the framework of the HS [8–10]. Their main conclusions are (evidence: B, agreement: 2) (i) standard treatment is based on steroids and VBL, (ii) Clinical response after the first 6 weeks of treatment is a good marker of further disease evolution. (iii) Prolonged treatment for at least 1 year reduces the risk of disease reactivations.

### Front Line Treatment and Evaluation of Response

Front line treatment of MS-LCH is based on the association of VBL 6 mg/m^2^ i.v. weekly bolus for 6 weeks, with prednisone 40 mg/m^2^/day given orally in three divided doses for 4 weeks and then tapered over the following 2 weeks. After the first 6 weeks of treatment, disease status should be reevaluated and treatment continued accordingly. The evaluation of the disease response is usually classified as “better” in case of complete resolution or regression of the disease, “worse” in case of progression of the disease, and “intermediate,” in case of stable or mixed response with new lesions in one site, and regression in another site. Other evaluation methods have been proposed such as the disease activity score [37].

In case of a good response (especially in the risk organs) but with some active disease still present in other sites, treatment with VBL, and steroids should be continued for another 6 weeks with: VBL 6 mg/m^2^ i.v. weekly bolus, and prednisone 40 mg/m^2^/day orally in three divided doses for 3 days every week. One or two intensive courses according to the above mentioned schedule should be followed by maintenance therapy for a total duration of up to 12 months with VBL 6 mg/m^2^ i.v. bolus every 3 weeks, and prednisone 40 mg/m^2^/day orally in three divided doses for 5 days every 3 weeks and 6 MP at a dose of 50 mg/m^2^/day is added if risk organ involvement is present.

### Second Line Therapy

Refractory disease in patients with hematological involvement or liver dysfunction is a rare but life-threatening situation [10, 38]. We suggest that such patients need to be referred to a specialized centre (evidence: D, agreement: 2). Therapeutic options (evidence: C) include combination chemotherapy with cladribine (2-CdA) and cytarabine (Ara-C) [39] or hematopoietic stem cell transplantation using reduced intensity conditioning regimen [40]. If there is evidence of disease progression in “non-risk organs,” treatment with 2-CdA as monotherapy [41] or even with further courses of a combination of VBL and steroids should be considered.

### Radiotherapy

Most experts in this field would no longer recommend radiotherapy due to the risk of long term sequelae, including the potential risk of developing a malignant tumor in the field of the radiotherapy [38]. However, there are some physicians who consider that radiotherapy may be useful for a single bone lesion in teenager (evidence: C, agreement: 2).

### Neurodegenerative Complications

Neurodegenerative complications represent a complex situation and such patients need to be managed by a multidisciplinary team. Several therapies have been attempted but with possibly occasional transitory responses. The treatment options include: retinoic acid (evidence: C) [42], combination treatment with vincristine and Ara-C (evidence: C) [43], intravenous immunoglobulin (evidence: D) [44], and cladribine (evidence: D) [45]. To date, intensive therapies have not shown any effect and should thus be avoided, especially as they may add to the morbidity.

### Monitoring and Supportive Care for Permanent Consequences

Although LCH is predominantly a benign and treatable disease, it can result in sequelae affecting various tissues involved [46]. Some may be present at diagnosis, while others may become manifest up to years and decades later. It is thus important to monitor these patients at least until growth is completed and possibly into adult life. The most common permanent consequences are endocrine, auditory, and orthopedic. Neurocognitive, pulmonary, and hepatic sequelae are rare but may cause significant morbidity. The recommended investigations and tests are shown in Table V. A scoring system for sequelae has been developed in order to observe the evolution and to standardize the recording of such problems [47].

**TABLE V:** Recommendations for Follow-Up of Patients With LCH After Diagnosis

Indication	Assesment
All patients	Routine assessment at clinically appropriate intervals including:
	History of thirst, polyuria
	Height, weight, pubertal status, neurological assesment
	FBC (CBC), ESR, Liver enzymes, Albumin
Bone involvement	X-ray oriented to the pathologic area at 6 weeks, 3 and 6 months and then depending on clinical findings
If vertebral involvement	Monitor for scoliosis especially during periods of rapid growth
If jaw involvement	Monitor dental development and jaw growth
Pulmonary involvement	Spirometry should be performed regularly (every 6–12 months) and if abnormal X-ray and high resolution computed tomography of chest may be needed
Endocrine involvement	
If endocrine signs and symptoms develops	See text for indications for endocrine testing and repeat depending on clinical findings and specialized advice
If proven hypothalamic-pituitary dysfunction	Head MRI, repeated after 1 year and then at 2, 4, 7, and 10 years
CNS involvement	
If neurological symptoms/signs develops	Neuropsychological tests, cerebellar function assessment and MRI of the head; repeat depending on clinical findings and specialized advice
If tumorous a lesion has been identified in the CNS	Repeat head MRI after 6 weeks (in symptomatic patients and those with tumorous lesions) and 3 months. Further images should be decided on the basis of the results of the first two examinations
If neurodegenerative findings on MRI, even without symptoms	Repeat head MRI is performed after 1 year and then at 2, 4, 7, and 10 years
Liver involvement	Consider ultrasound scan/MRI of liver or cholangiography and repeat depending on clinical findings and specialized advice
Ear/temporal bone involvement	Audiogram at end of treatment and reassessed at start of school and if any new symptoms develop

CT, computed tomography; MRI, magnetic resonance imaging, CBC, complete blood count; ESR, erytrocyte sedimentation rate; CNS, central nervous system; DI, diabetes insipidus, ENT: ear nose throat; BAL, broncho-alveolar lavage, GH, growth hormone.

#### Endocrine complications

DI is the most frequent endocrinopathy associated with LCH with a frequency from 15% to 30% of cases [30]. It is thus important to investigate thirst and polyuria in LCH patients, even many years after the diagnosis of LCH.

Growth hormone deficiency is the most frequent anterior pituitary hormone loss and occurs in up to 10% of patients. Measurement of height and weight and assessment of puberty is therefore recommended every 6 months or 1 year until growth is completed. Any child whose growth is below that expected may need to be investigated as suggested by the consensus guidelines of the GH Research Society [48].

Other hormone deficiencies may occur. These include delayed puberty and rarely panhypopituitarism. Puberty should be assessed according to Tanner stages and need investigation in the following cases: delayed onset of puberty (B2 >13 years in girls, P2, T2 >14 years in boys), delayed onset of period in girls (>14 years), precocious puberty (B2 <8 years in girls, P2, T2 <9 years in boys), arrest, or regression of pubertal development.

In case of delayed growth/puberty, bone age should be assessed by X-ray, and anterior pituitary function tests should be performed to assess secretion of GH, LH, FSH, ACTH, and thyroid function. If hormone deficiency is confirmed by the stimulation tests, MRI scan of head (Appendix 1) should be performed. Bone mineral density (DEXA) scan needs to be monitored in patients with GH deficiency, delayed puberty, or panhypopituitarism.

#### Orthopedic

When several vertebrae are affected, scoliosis may become manifest later in life, in particular during periods of rapid growth such as puberty. Children should be assessed clinically at least annually in order to identify any early signs of scoliosis. They should be referred to the orthopedic surgeon in order to start preventive physical therapies (e.g., orthopedic corset/brace or neck collar) in order to manage this proactively. If facial bones are affected, facial asymmetry may become manifest and reconstructive surgery may be required.

##### Hearing

Subjects with involvement of the middle or inner ear and the temporal bone should be monitored with audiometry at diagnosis and at end of treatment and reassessed at start of school and if any new symptoms develop. Early diagnosis and interventional strategies such as hearing aids can avoid deterioration of school performance and significantly improve learning outcome.

##### Oral tissue and jaw

Children with involvement of gums and jaw should be monitored for dental development and growth of the jaw.

##### Neurological

Children with multisystem LCH should be regularly followed up clinically since they are at risk of developing late neuropsychological sequelae, in particular cerebellar ataxia and learning difficulties. In children with relevant history and/or abnormal neurological examination, further investigations including neuropsychological tests, cerebellar function assessment [49, 50], and MRI of the head as described in Appendix 1 should be performed.

##### Lungs

In those with a history of lung involvement, spirometry should be performed regularly and if abnormal or progressive, X-ray and computed tomography of chest may be needed. The dangers of smoking should be explained and smoking avoided. Pulmonary involvement may also lead to respiratory insufficiency due to fibrosis and emphysema.

##### Liver

Liver involvement is rare, but can cause serious morbidity. In those with abnormal liver function consider ultrasound scan, MRI of liver, or cholangiography as clinically indicated. A subset of young children with liver involvement may subsequently develop sclerosing cholangitis that progresses to cirrhosis; treatment for these children includes liver transplantation.

### Associated Malignancies

There is a recognized association between LCH and malignancies [17]. The malignancies may precede, occur concurrently or follow the diagnosis of LCH and should be considered at every clinical visit. Acute lymphoblastic leukemia and lymphoma more often occur prior to the diagnosis of LCH but may be diagnosed within 5 years after LCH. Myeloid leukemias usually follow LCH especially in those patients exposed to etoposide, alkylating agents and/or radiotherapy. Solid tumors may occur concurrently or follow the diagnosis of LCH. Most of those that followed LCH developed in a previous radiation field. With the current treatment strategies it is expected that these types of secondary malignancy will be rare. Patients treated with radiotherapy should see their doctor in case of symptoms involving the irradiated area.

### Follow-Up/Duration and Frequency

Recommendations for follow-up are shown in Table V. They are inspired by the long term follow-up for childhood cancer survivors [51, 52]. Every patient should be followed by the local physician and if at any time a particular issue needs to be addressed, referral to a specialist is recommended. All patients should be followed for a sufficient time period, defined as (i) at least 5 years after the end of therapy; or (ii) 5 years after the last disease reactivation, in those who did not receive systemic therapy; or (iii) until final growth and pubertal development have occurred.

#### Perspectives

LCH is a rare disease potentially resulting in death or permanent sequelae. The burden of therapy may also be extremely heavy. There is an obvious need for a full assessment of each patient with a rational treatment tailored to the risks of the individual patient, which contributes to further fundamental and clinical research in this field.

In 2010, Badalian-Very et al. [53], reported somatic mutations of the BRAF oncogene in about half of the LCH patients in their series, and this finding was recently confirmed by other teams [54, 55]. This discovery may have a significant potential impact if we consider the possibility of treating LCH with the new class of BRAF inhibitors. However, this promising discovery will need to be verified and concretized before these drugs can be used for treatment of LCH. The group(s) of LCH patients who may benefit from BRAF inhibitor treatment must be determined and balanced with toxicities as in the case of melanoma [56, 57]. Knowledge about drug schedule and safety, especially long-term effects [57] and mechanisms of resistance [58] must be acquired.

Progress may be expected from collaborations organized at national and international levels, among specialist groups and expert networks. Collection of tissue and blood samples in biobanks is essential for improving the understanding of the biology of this rare and fascinating condition. New international protocols will soon be opened and continue to represent an opportunity to develop global research in LCH (see http://www.histiocytesociety.org and http://www.histio.net).

##### Euro Histio Net Partners—Institutions

Euro Histio Net is a project funded by the European Commission/DG Sanco and associated both medical institutions and patients associations as listed below. 1 Reference Centre for Histiocytosis, Hopital Trousseau, Assistance Publique – Hopitaux de Paris, France. Jean Donadieu, MD, PhD, Coordinator, Abdelatif Tazi, MD, PhD, Jean François Emile, MD, PhD, Milen Minkov, MD, PhD, Associated Partners, 2 Hospital Universitario Cruces Barakaldo, Spain. Itziar Astigarraga, MD, Associated Partner, 3 Children's Cancer Research Institute, St. Anna Children's Hospital, Vienna, Austria. 4 Istituto G. Gaslini, Genova, Italy. Riccardo Haupt, MD, Associated Partner, 5 Johns Hopkins, Baltimore, Maryland, USA. Robert J. Arceci, MD, PhD, Collaborating Partner, 6 Azienda Ospedaliero-Universitaria Meyer, Firenze, Italy. Maurizio Aricò, MD, PhD, Collaborating Partner, 7 Elisabethinen Hospital, Linz, Austria. Michael Girschikofsky, MD, Collaborating Partner, 8 Karolinska Institutet, Stockholm, Sweden. Jan-Inge Henter, MD, PhD, Collaborating Partner, 9 Children's University Hospital, Hamburg, Germany. Gritta Janka, MD, PhD, Collaborating Partner, 10 Hotel Dieu de France Hospital, Beirut, Lebanon. Claudia Djambas Khayat, MD, Collaborating Partner, 11 Hellenic Air Force & Veterans General Hospital, Athens, Greece. Polyzois Makras, MD, PhD, Collaborating Partner, 12 Hospital de Clínicas de Porto Alegre, Brasil. Mariana Michalowski, MD, Collaborating Partner, 13 Watford General Hospital, Watford, UK. Vasanta Nanduri, MD, Collaborating Partner, 14 Dana Farber Cancer Institute and Childrens Hospital, Boston, Massachusetts, USA. Carlos Rodriguez-Galindo, MD, Collaborating Partner, 15 Medical University Plovdiv, Bulgaria. Mariya Spasova, MD, PhD, Associate Professor, Collaborating Partner, 16 University Pediatric Hospital, Lublin, Poland. Maria Jolanta Stefaniak, MD, PhD, Collaborating Partner, 17 UZ Leuven, Leuven, Belgium. Stefaan Van Gool, MD, PhD, Collaborating Partner, 18 University Hospitals of Leicester, Leicester Royal Infirmary Childrens Hospital, Leicester, United Kingdom. Johann Visser, MD, Collaborating Partner, 19 Hospital for Sick Children, Toronto, Ontario, Canada. Sheila Weitzman, MD, Collaborating Partner, 20 Newcastle University, Newcastle, United Kingdom. Kevin Patrick Windebank, MD, Collaborating Partner, 21 Clinique Al Madina, Casablanca, Morocco. Saadia Zafad, MD, PhD, Collaborating Partner.

##### Euro Histio Net Partners—Patient Associations

1 The Histiocytosis Research Trust, United Kingdom, http://www.hrtrust.org Associated Partner; 2 Artemis Association on Histiocytoses, Greece, http://www.histioartemis.gr Collaborating Partner; 3 Asociacion Espanola contra la Histiocitosis de celulas de Langerhans (ACHE), Spain, http://www.histiocitosis.org Collaborating Partner; 4 Association Histiocytose France (A.H.F.), France, http://www.histiocytose.org Collaborating Partner; 5 Associazione italiana ricerca istiocitosi (AIRI LCH onlus), Italy, http://www.istiocitosi.org Collaborating Partner; 6 Erwachsenen Histiozytose X e.V (EHX e.V.)., Germany, http://www.histiozytose.com Collaborating Partner; 7 Föräldraföreningen För Histiocytos (Ffh), Sweden, http://www.histiocytos.se Collaborating Partner; 8 Histiocytosis Association of America (HAA), USA, http://www.histio.org Collaborating Partner; 9 Histiozytosehilfe e.V., Germany, http://www.histiozytose.org Collaborating Partner; 10 LCH-Belgium, Belgium, http://www.lch.be Collaborating Partner; 11 The ECD Global Alliance, USA, http://www.erdheim-chester.org Collaborating Partner.

## Abbreviations

LCH: Langerhans cell histiocytosis
MRI: magnetic resonnance imaging
CNS: central nervous system
DI: diabetes insipidus
MS-LCH: multi system Langerhans cell histiocytosis
SS-LCH: single system Langerhans cell histiocytosis
RO: risk organs.
