# Supplementary material for: Langerhans Cell Histiocytosis (LCH): Guidelines for Diagnosis, Clinical Work-Up, and Treatment for Patients Till the Age of 18 Years
Source: Pediatr Blood Cancer. 2012 Oct 25;60(2):175–84. doi: 10.1002/pbc.24367 (PMC4557042; doi:10.1002/pbc.24367)
Supplement: Supplementary file 1 — Supplementary Appendix [file pbc0060-0175-sd1.doc]

Appendix 1

## Head MRI in LCH Patients

**Technique.** The MRI examination protocol must collect information sufficient to evaluate the entire brain, particularly the hypothalamus-pituitary axis, and also all craniofacial bones. Its aim is to systematically seek any neuro-degenerative involvement and/or tumorous lesion and meningeal involvement [59, 60]. The use of intravenous contrast (Gadolinium chelates) is mandatory. The following protocol is recommended: Axial and sagittal T1w. slices of the entire brain, fine T1 w. sagittal slices focused on the pituitary gland (3 mm / 0.3 mm or below), axial T2W. and FLAIR w. slices (except age < 1 year) of the entire brain. It is not recommended to use the option “magnetization transfer contrast” (MTC). If it is however performed, the same technique has to be used every time and this information has to be specified on the report. After injection of Gadolinium the MRI scan needs to be repeated according to data obtained on the first series by (T1 w. slices), fine sagittal slices of the pituitary and coronal slices of the brain. Additional sequences may be taken if indicated.

**Frequency:** If a lesion has been identified in the CNS it is suggested to repeat the examination after 6 weeks (in symptomatic patients and those with tumorous lesions) and 3 months. Further images should be decided on the basis of the results of the first two examinations. In case of clinical hypothalamic dysfunction or neurodegenerative findings on MRI, even without symptoms, it is suggested to perform a second MRI after one year and then at 2, 4, 7, and 10 years. This recommendation is not ‘evidence based’ but is consensual (class of agreement 1) . If after 10 years there is no clinical deterioration, further MRI is recommended only upon clinical indication.
